# Radiographic Enlargement of Mandibular Canal as an Extranodal Primary Non-Hodgkin's Lymphoma Early Sign in an Asymptomatic Patient

**DOI:** 10.1155/2017/9193165

**Published:** 2017-02-19

**Authors:** Luciana Munhoz, Felipe Pereira Marcos Marsan, Emiko Saito Arita

**Affiliations:** ^1^Department of Stomatology, School of Dentistry, University of São Paulo, 2227 Lineu Prestes Avenue, 05508-000 São Paulo, SP, Brazil; ^2^Department of Odontology, University of São Paulo, 448-475 Cesário Galeno Street, 03071-000 São Paulo, SP, Brazil

## Abstract

Non-Hodgkin's lymphoma (NHL) is a lymphoproliferative disorder, from a subgroup of heterogeneous hematologic malignancies; the term “extranodal” refers to malignant involvement of tissues other than lymph nodes, tonsils, spleen, pharyngeal lymphatic ring, or thymus. Only 0.6% of all NHL are at mandible alone, and it may involve the inferior alveolar canal. We describe a case of bilateral enlargement of the mandibular canal without symptomatology, which was shown in a panoramic radiograph and cone beam computed tomography in a rehabilitation routine exam, as an early sign of primary extranodal NHL.

## 1. Introduction

Non-Hodgkin's lymphoma (NHL) is a lymphoproliferative disorder, from a subgroup of heterogeneous hematologic malignancies that also includes other three different lymphoproliferative disorders: Hodgkin disease, lymphocytic leukemia, and multiple myeloma [1]. The term “extranodal” refers to malignant involvement of tissues other than lymph nodes, tonsils, spleen, pharyngeal lymphatic ring, or thymus [1].

Approximately 20% to 30% of NHL occur at extranodal sites [2]; at head and neck the most common involvement of extranodal NHL is at sinonasal site [3]. When NHL affects oral cavity, around only 15% to 45% arise in maxilla or mandible [4]. The most usual sites are maxilla, mandible, palatal soft tissue, and gum, respectively [5]. Only 0.6% of all NHL are at mandible alone [5], and it may involve the inferior alveolar canal, frequently without any radiographic sign of bony changes [6].

In oral cavity, extranodal sites of NHL may be found as solid masses of spongy consistency [7, 8]. Bone marrow infiltration by malignant lymphomas is influenced by the characteristic pattern according to the lymphoma subtyping [9], and these malignant patters may provide diagnostic hints for NHL subtyping [10].

Considering head and neck sites, primary NHL occurs more often in male patients than female [11, 12], in the fifth to seventh decades of life [11, 12]. Male Caucasians patients are more often affected than other genders [11–13].

In radiographic findings, enlargement of inferior alveolar canal is extremely unusual, and it is often related to malformations or benign lesions [14, 15]. The presence of mandibular canal widening in extranodal NHL patients is even rarer; primary extranodal NHL frequently arises in the medullar cavity of single long bones [13]. A review of English language medical literature using Pubmed database from 1990 to 2016 reveled only four previous reports of NHL associated with mandibular canal enlargement [13, 14, 16, 17]. In the present report, we describe a case of bilateral enlargement of the mandibular canal without symptomatology, which was shown in a panoramic radiograph and cone beam computed tomography in a rehabilitation routine exam.

## 2. Case Report

### 2.1. Case History and Clinical Findings

Male Caucasian patient, 39-year-old, was referred to a private Radiologic Clinic in São Paulo, Brazil, for radiographic exams with the purpose of planning oral rehabilitation. At the moment of evaluation, the patient did not report any clinical symptoms or showed clinical signs of intraoral or extraoral alterations. The systemic health history did not provide relevant information, and the patient denied carrying any syndrome or having knowledge of any carrier relative.

### 2.2. Imaging Evaluations

**Figure 1:**
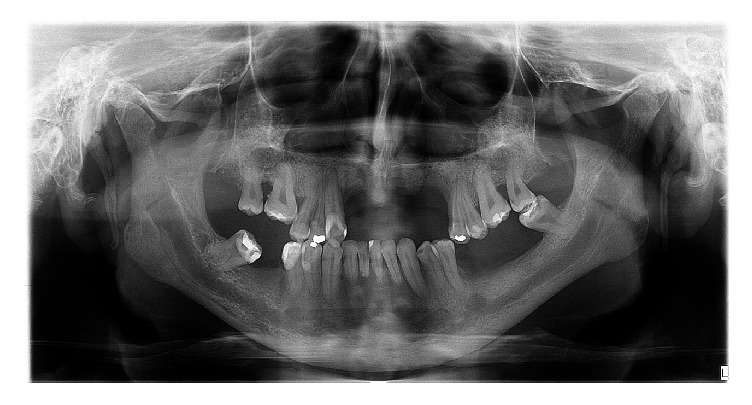
Panoramic Radiograph of the case. Enlargement of mandibular canal in both sides.

**Figure 2:**
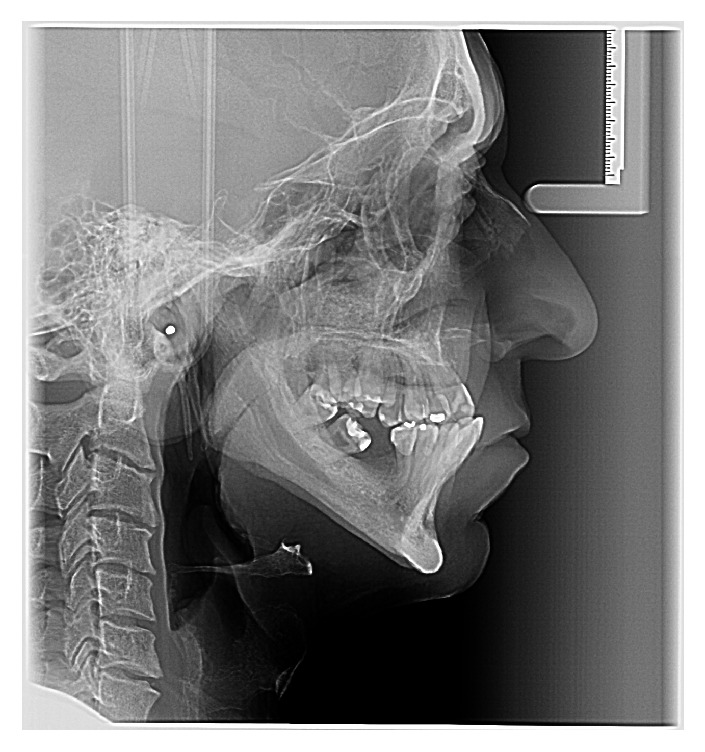
Teleradiograph; the enlargement is also observed.

The panoramic radiograph showed the absence of 11 teeth (4 at mandible), alveolar bone loss, trabecular bone with increased thickening as well as mandibular cortical erosion (indicating reduction of bone mineral density), and elongated styloid process. The mandibular canal and mental foramen presented inferior-superior enlargement, bilaterally (Figure 1). The lateral teleradiography also demonstrated the mandibular canal enlargement, beginning in the retromolar trigone and affecting the mandibular ramus (Figure 2).

**Figure 3:**
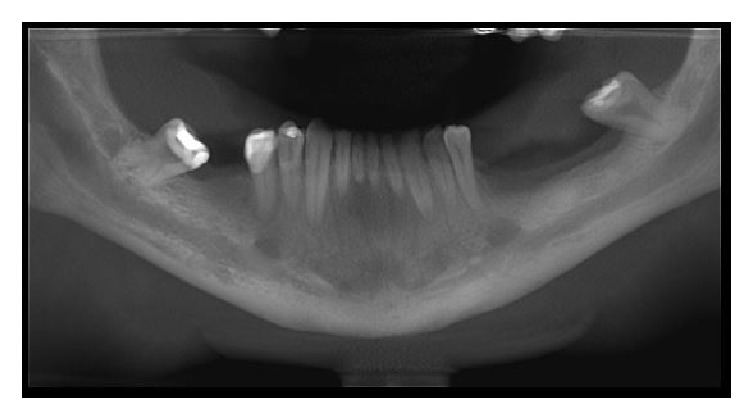
CBCT panoramic slice.

**Figure 4:**
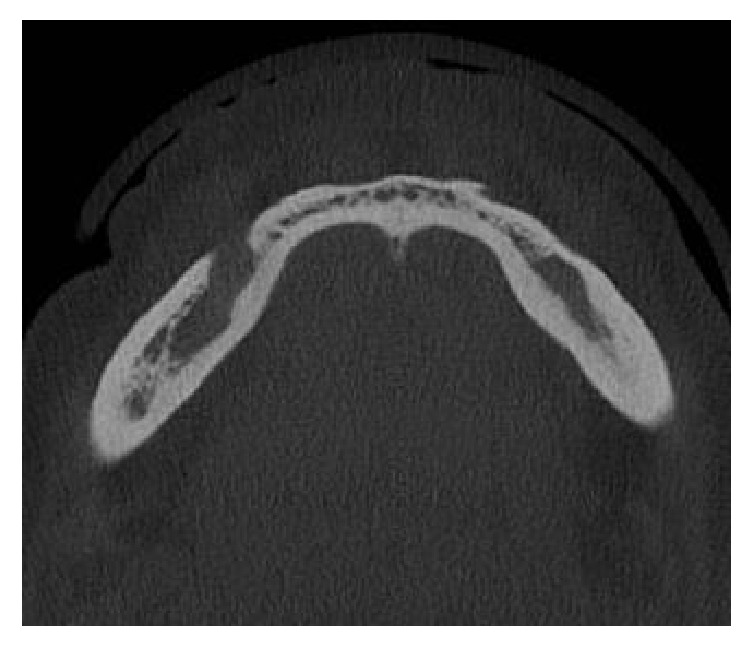
CBCT axial slice demonstrating rupture of the mandibular cortex.

**Figure 5:**
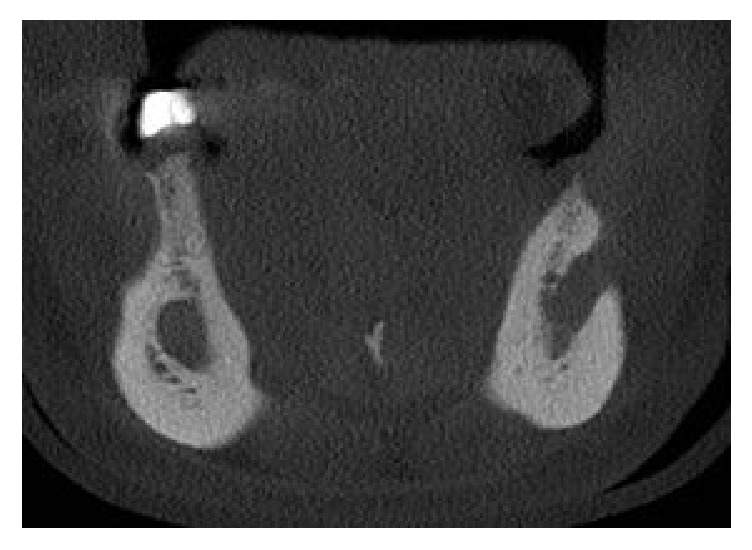
Frontal slice; enlargement of mental foramen.

In the cone beam computed tomography (CBCT), multiplanar reconstruction demonstrated marked increase in the diameter of the mandibular canal throughout their length, on both sides, as well as the enlargement of mental foramens. Right and left side mandibular canal's bone cortices were preserved but they were thinning. At the right side, in axial and coronal sections, foramen's bone cortex exhibited discontinuity at medial wall. The radiolucent unilocular fusiform areas apparently were unconnected with the teeth or root tips (Figures 3, 4, and 5).

The initial diagnostic hypotheses were bilateral hemangioma, malignant lymphangiona [18], any related syndrome that would affect neural sheath Schwann cells, like neurofibromatosis [19, 20]; multiple endocrine neoplasia type 2b with bilateral involvement and bifurcated inferior alveolar canals [21], arteriovenous malformation [22], vascular leiomyoma associated with mandibular canal [23], and extranodal NHL [14].

The final diagnosis was provided by the histopathological examination and hematologic examinations.

## 3. Discussion

A better understanding of the clinical and imaging features of this type of lesion is necessary to avoid diagnostic confusion, especially with benign alterations or vascular malformations with involvement of multiple sites. Previous reports on lymphoma demonstrated a single expansion of the bone, with no bone destruction [24, 25]. A classical but not frequent [17, 26] radiologic finding in head and neck lymphoma is ill defined or lytic destruction, suggestive of malignant neoplasms or osteomyelitis [11]; however, the imaging features of the present case resemble a benign lesion more than a malignancy. Probably, as described by the two latest mandibular canal extranodal NHL report [13, 14], the lymphoma encroached so slowly into mandibular canal such that surrounding bone did not lose its characteristic of subtle sclerosis that marks the canal wall; and the limits seen were well defined.

The patient of the present case reported was asymptomatic, with primary complaint of teeth absence. In spite of this finding, clinical presentation of NHL extranodal bone lesions usually includes history of swelling, pain, paresthesia, or hyperesthesia along alveolar nerve extension and distribution, as well as lymphadenopathy [11]. Hyperesthesia may be related to compression or infiltration of the inferior alveolar nerve [24]. Other unspecific symptoms, such as tooth mobility, may refer to dental abscess or osteomyelitis [27, 28], especially to lymphomas at alveolar process, which are confounders to the NHL diagnostic [13].

Establishing the diagnostic hypothesis based on routine X-rays techniques, such as panoramic and lateral teleradiography, is quite difficult, especially in the absence of local and systemic clinical alterations or further accurate patient's clinical history information. However, panoramic images are considered an important tool of investigation at early stage of malignancies like NHL, due to the fact that it allows the professionals to detect and visualize the first signs of the disease. CT may confirm these findings [5].

In the present case, panoramic radiograph was crucial to initiating deep imaging investigations due to the evident radiolucent fusiform lesion with notch-like margins and with no bony septae at inferior alveolar canal especially because of the asymptomatic characteristic presented by the patient. Notwithstanding, compared with standard x-ray techniques, MRI and CTC scans provide much more valuable information for diagnostic hypothesis postulation and preoperative planning [15].

On computed tomography (CT) scans, beyond the expansion of the mandibular canal path walls, erosion of the cortex of mandibular canal should be studied, as well as other radiographic signs, such as thinning or disruption of bone cortices associated with the neoplasms. Magnetic resonance imaging (MRI) findings, unfortunately, have not been reported to our knowledge. MRI findings would help to differentiate NHL from solid purely cystic lesions, before the histopathological exam.

The bilateral presence of the neoplasm has raised questions as to whether the patient is carrying syndromes, genetic disorders, or even vascular malformations. The differential diagnostic hypothesis first included neurofibromatosis, due to the particular aspect of widening of the mandibular canal [19, 20]. Other hypotheses were multiple endocrine neoplasia type 2b [21] with bilateral mandibular canal involvement and arteriovenous malformation [22]. Unilateral or localized intramandibular canal lesions such as solitary neurofibromas [29, 30], traumatic neuroma of the inferior alveolar nerve [31], localized hypertrophic neuropathy (intraneural perineurioma) [32], vascular leiomyoma [23], and schwannomas were excluded [15] after CT examination. Extranodal NHL was not considered at first, due to its frequency at mandible, or even malignant pathologies, because of the radiographic features.

Although rich information was provided by radiological examination, the definitive diagnosis was reached through histopathological exam followed by hematological studies. Immunohistochemical phenotyping is also applied for NHL [13]. The prognosis is determined by clinical staging and histological grade; primary extranodal NHL progression may also lead to a fatal outcome [13].

The recommended treatment for intraosseous lymphomas of the jaws may include nonconservative surgery, although surgical eradication is not the first choice of treatment; frequently chemotherapy or radiation or both are used [12].

Thus, regarding the features of intraosseous NHL of inferior alveolar nerve on plain radiographs and CBCT, it is important to consider this NHL as a diagnostic hypothesis to benign tumors, especially when it is at a bilateral site, despite its rarity. Due the severity of the disease and the possibility of a fatal outcome, it is important to avoid delaying to definitive diagnosis.
